# Fed State Prior to Hemorrhagic Shock and Polytrauma in a Porcine Model Results in Altered Liver Transcriptomic Response

**DOI:** 10.1371/journal.pone.0100088

**Published:** 2014-06-17

**Authors:** Charles Determan, Rebecca Anderson, Aaron Becker, Nancy Witowski, Elizabeth Lusczek, Kristine Mulier, Greg J. Beilman

**Affiliations:** Department of Surgery, Division of Critical Care and Acute Care Surgery, University of Minnesota, Minneapolis, Minnesota, United States of America; Georgia Regents University, United States of America

## Abstract

Hemorrhagic shock is a leading cause of trauma-related mortality in both civilian and military settings. Resuscitation often results in reperfusion injury and survivors are susceptible to developing multiple organ failure (MOF). The impact of fed state on the overall response to shock and resuscitation has been explored in some murine models but few clinically relevant large animal models. We have previously used metabolomics to establish that the fed state results in a different metabolic response in the porcine liver following hemorrhagic shock and resuscitation. In this study, we used our clinically relevant model of hemorrhagic shock and polytrauma and the Illumina HiSeq platform to determine if the liver transcriptomic response is also altered with respect to fed state. Functional analysis of the response to shock and resuscitation confirmed several typical responses including carbohydrate metabolism, cytokine inflammation, decreased cholesterol synthesis, and apoptosis. Our findings also suggest that the fasting state, relative to a carbohydrate prefed state, displays decreased carbohydrate metabolism, increased cytoskeleton reorganization and decreased inflammation in response to hemorrhagic shock and reperfusion. Evidence suggests that this is a consequence of a shrunken, catabolic state of the liver cells which provides an anti-inflammatory condition that partially mitigates hepatocellar damage.

## Introduction

Hemorrhagic shock is a leading cause of trauma-related mortality in both civilian and military settings. Efforts over the years have significantly improved survival in the military sector [1]; however, hemorrhagic shock from traumatic injury results in multiple alterations in the metabolic state of an organism many of which are not fully elucidated. Civilian data estimates between 1400 and 14,000 preventable hemorrhagic trauma deaths occur per year in the United States [2]. Military data has consistently reported hemorrhagic shock as the leading cause of preventable deaths [3]–[5].

Hemorrhagic shock results in inadequate tissue perfusion leading to decreased oxygen availability to mitochondria. This condition causes a switch towards anaerobic metabolism in addition to a complex inflammatory response. The liver serves an important function as a regulator of metabolism during stressed states. Initially, the shift towards anaerobic metabolism stimulates the liver to increase glycogenolysis and process elevated lactate produced in the peripheral tissues. The liver also provides a major site of detoxification and production of alternate metabolic fuel sources including amino acids and lipids.

Previous research in our laboratory revealed increased acute lung injury and multiple organ failure with a trend toward increased mortality in pigs receiving a carbohydrate diet prior to hemorrhagic shock relative to fasted pigs [6]. Our previous metabolomics research has reported an altered response to fed state in the liver and urine as well as phase of care in serum following hemorrhagic shock and resuscitation [7]–[9]. The objective of this study was to determine if this differential response is related to varied genetic expression in the liver with the hypothesis that there would be quantifiable differences in liver mRNA expression reflecting an altered response to shock and resuscitation with respect to fed state. To test this hypothesis, we used our well-established model of hemorrhagic shock and polytrauma comparing the effect of providing a carbohydrate (CPF) versus a fasted (FS) diet prior to insult. Extraction of mRNA from liver biopsies and subsequent RNA-Sequencing was used to compare between CPF and FS animals at four timepoints: Baseline (B), and 2, 8 and 20 hours after resuscitation (FR2, FR8, and FR20). Secondly, livers samples were examined over the course of polytrauma, hemorrhagic shock and resuscitation within each group to determine how mRNA expression changed within each group.

## Materials and Methods

### Animals

Male Yorkshire-Landrace pigs (15–20 kg) were purchased from Manthei Hog Farm, LLC (Elk River, MN) and housed in Research Animal Resources (RAR) at the University of Minnesota. All studies were approved by the University of Minnesota Institutional Animal Care and Use Committee (IACUC). Pigs were fasted overnight prior to surgery, but were allowed water *ad libitum*. All surgeries were performed under anesthesia and all efforts were made to minimize suffering.

### Animal Preparation and Hemorrhagic Shock Protocol

**Figure 1:**
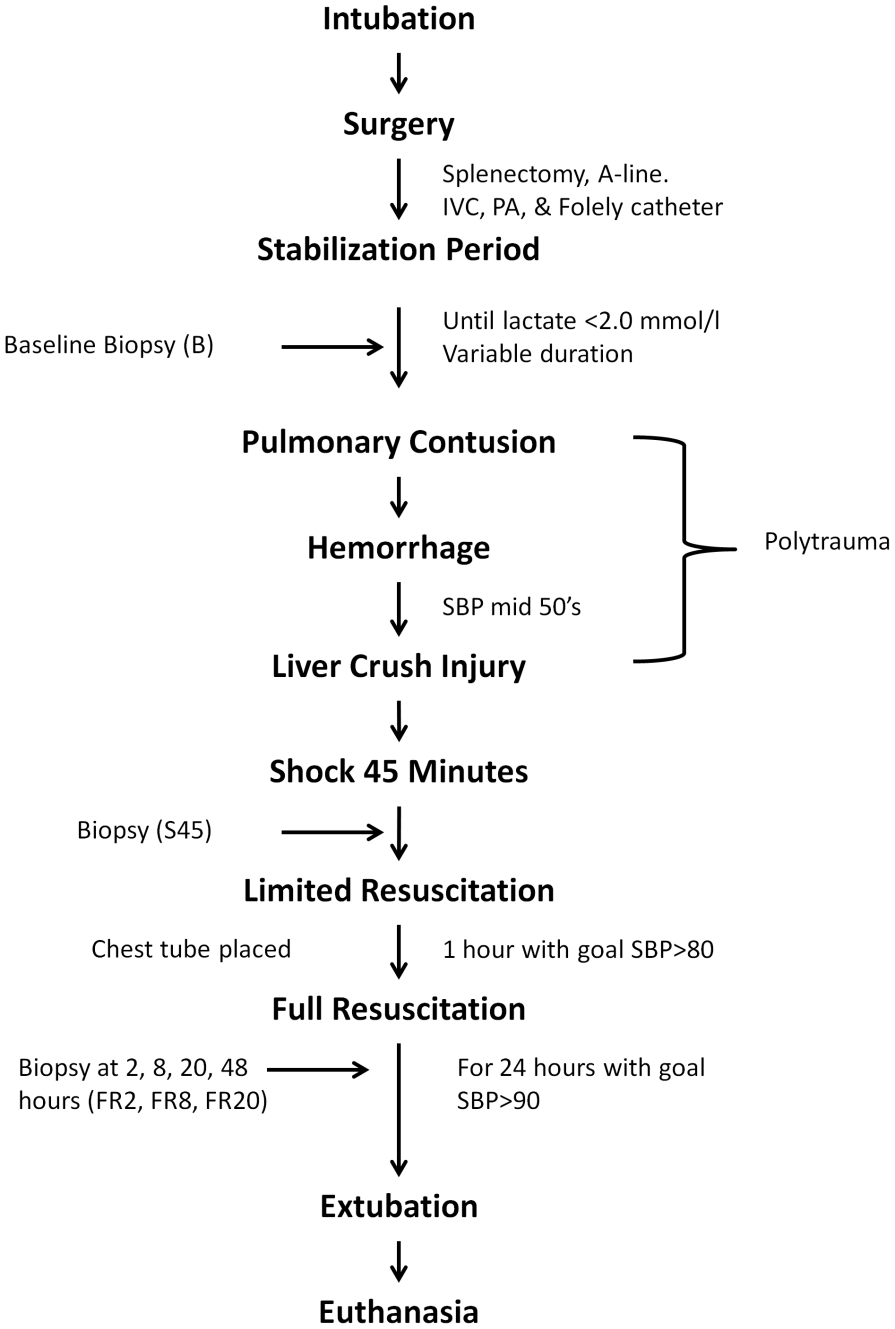
Graphical representation of the experimental timeline described in Methods.

Sixty four (64) juvenile, male Yorkshire pigs were used in this study. All animals were fasted overnight. Two experimental groups were utilized: Carbohydrate Prefed (CPF, n = 32) and Fasted (FS, n = 32). CPF animals were given 7cc/kg bolus of Karo Syrup (mixture of sugars including ∼ 15% glucose, maltose, fructose and sucrose) diluted with water 1 hour prior to induction. The full experimental polytrauma and shock protocols have been described in detail previously [9], [10]. Briefly, animals were instrumented and splenectomized. Polytrauma was induced by a captive bolt device to create a blunt percussive injury to the chest followed by hemorrhage and a liver crush injury using a Holcomb clamp technique [11]. Hemorrhagic shock was induced by withdrawal of blood from the inferior vena cava until a systolic pressure of 45 to 55 mmHg was reached for 45 minutes (S45) to simulate time prior to medical help. Typically, this resulted in withdrawal of approximately 40% of the pig’s blood volume. Blood was placed in an acid-citrate-dextrose bag for later use. Following the shock period, animals received lactated Ringer’s fluid given as 20 cc/kg intravenous (IV) boluses to maintain a systolic blood pressure greater than 80 mmHg for one hour of limited resuscitation to simulate transportation to a medical center; then underwent full resuscitation protocol for the following 24 hours (Figure 1). This resuscitation included fluid, shed blood, and ventilator support per our standard protocol. After the resuscitation period, animals were extubated and sent to recovery and subsequently euthanized.

At several time points throughout the experiment, liver biopsies were taken from the periphery of the liver ranging in weight from 0.2 to 0.6 grams. Biopsies were flash frozen in liquid nitrogen and stored at −80°C until preparation for RNA extraction. Biopsies were taken at the following timepoints: baseline after the animal stabilized from instrumentation (B), 45 minutes after hemorrhage (S45), 2, 8, and 20 hours after full resuscitation (FR2, FR8, FR20). RNA extractions and sequencing were conducted on B, FR2, FR8, and FR20. Analysis was not conducted on the S45 timepoint because of the short time period from baseline to allow RNA changes to take place.

### RNA Preparation and Sequencing

#### RNA extraction and quality assessment

In a previous study, some of the samples from the original 64 animals had been utilized. As such, only those that had all consecutive timepoints were included. Ultimately, only 18 Fasted animals and 5 Prefed animals were analyzed at each timepoint (total n = 92). RNA was purified from liver samples using QIAshredder and Qiagen RNeasy Mini Kits (Qiagen, Chatsworth, CA). Total RNA isolates were quantified using a fluorimetric RiboGreen assay. Total RNA integrity was assessed using capillary electrophoresis, generating an RNA Integrity Number (RIN). All of the samples were verified as high quality (>1 microgram, RIN = 8+) and thus were converted to Illumina sequencing libraries.

#### Library creation

RNA samples were converted to sequencing libraries using Illumina’s Truseq RNA Sample Preparation Kit (RS-122-2001). In brief, 1 microgram of total RNA was enriched for mRNA using oligo-dT coated magnetic beads, fragmented, and reverse transcribed in cDNA. The cDNA was fragmented into smaller pieces blunt-ended, and ligated to indexed adaptors and amplified using 15 cycles of PCR. Final library size distribution was validated using capillary electrophoresis and quantified using PicoGreen fluorimetry and qPCR. Libraries were successfully sequenced for all samples.

#### Cluster generation and sequencing

Truseq libraries were hybridized to a paired-end flow cell and individual fragments were clonally amplified by bridge amplification on the Illumina cBot. Libraries were clustered at a concentration of 12 pM. The flow cell was then loaded on the HiSeq 2000 and sequenced using Illumina’s Sequencing by Synthesis (SBS) chemistry. Upon completion of a read, a 7 base pair index was performed for sample identification. Samples were run for 100 cycles with 10 million single reads per sample.

#### Primary analysis and de-multiplexing

Base call (.bcl) files for each cycle of sequencing were generated by Illumina Real Time Analysis software. The base call files and run folders were then exported to servers maintained at the Minnesota Supercomputing Institute (Minneapolis, MN). Primary analysis and de-muliplexing were performed using Illumina’s CASAVA software (version 1.8.2) resulting in FASTQ files.

### Bioinformatics and Data Analysis

RNA-Seq read sequences produced by the Illumina HiSeq 2000 were aligned with the TopHat software [12] to the NCBI Sscrofa 10.2 reference genome. The BAM files from the TopHat mapping were sorted using SAMtools [13] and raw counts estimated by the Python script HTSeq count (http://www.-huber.embl.de/users/anders/HTSeq/) using the NCBI Sscrofa 10.2 reference genome. Identified contigs were converted to human orthologs with the BiomaRt Bioconductor package [14] which facilitates access to BioMart annotation resources [15]. Gene names used for identification are the official Human Genome Organization (HUGO) Gene Nomenclature Committee (HGNC) designations.

The resulting raw counts per gene were used by EdgeR [16] to estimate differential expression (DE). EdgeR (Bioconductor release 3.2.4) uses a pair-wise design to measure differential expression. The analysis is based on a negative binomial model that uses over-dispersion estimates to account for biological variability (i.e., sample to sample differences); this is an alternative to the Poisson estimates of biological variability that are often inappropriate [17]. Genes with less than 10 reads were excluded from the analysis and TMM normalization of the sequenced libraries was performed to remove effects due to differences in library size [18]. The most stringent dispersion method (tag-wise) was used to ensure that differential expression was not due to individual differences. EdgeR generates a log2 fold change for each gene, p values and the Benjamini-Hochberg false discovery rate (FDR) are calculated to statistically test the measured DE. Lastly, genes were filtered to those reporting log2 fold changes ≥1 or ≤ −1 (i.e. 2 fold change). The complete R code and parameters used for the analysis are provided in the Supplementary Material (Supplementary File 7).

### Gene Ontology Analysis

The differentially expressed transcripts calculated between fed states at each time point (e.g. FSvCPF, FR2) and between timepoints within each group (e.g. FR8vB, FS) were analyzed using the functional annotation tools of DAVID [19], [20] and additional literature searches. Lists of DEGs from each comparison were entered into DAVID in addition to the complete list of sequenced genes to serve as background for analysis. DAVID analysis provides both functional annotations of each gene (Functional Annotation Table) and of each list of genes (Functional Annotation Clustering) to provide functional information using gene ontology [21]. The Functional Annotation Clustering tool provides an overall picture of the overrepresented and enriched functions by consolidating redundancies in gene ontology categories. Information on specific genes was obtained using the Functional Annotation Table tool and the scientific literature.

## Results

### RNA-Sequencing Quality

The RNA-sequencing (RNA-seq) results from the Illumina HiSeq 2000 were output as FASTQ format and mapped to the NCBI Sscrofa 10.2 release of the pig genome (ftp://ftp.ncbi.nih.gov/genbank/genomes/Eukaryotes/vertebrates_mammals/Sus_scrofa/Sscrofa10.2/) using TopHat [12]. One FR8 sample failed to sequence successfully and was omitted from further analysis. For each sample, approximately 11.8 million reads were generated (range, 6.9 to 20.6 million reads). On average 88.8% of the reads generated were mapped uniquely (range, 85.7–98.3 percent mapped). Following conversion to human orthologs and Human Gene Nomenclature Committee (HGNC) notation, to facilitate functional analysis, we identified 16,498 genes. The raw data has been made available through the Gene Expression Omnibus database accession number GSE55674.

### Differentially Expression

We initially compared gene expression differences between CPF and FS animals at Baseline. Of the 16,498 genes, 68 were identified as differentially expressed genes (DEGs) with 14 upregulated and 54 downregulated in CPF relative to FS. In addition, subsequent analysis comparing each timepoint relative to baseline between CPF and FS identified 13, 29, and 30 DEGs at FR2, FR8 and FR20 versus baseline respectively. At each time point following resuscitation, all DEGs were higher in CPF compared to FS except 2 genes at FR20 versus baseline (CHKA and GCK) (Supplementary File 1).

We then compared gene expression differences between each time point and baseline within each fed state. Within carbohydrate prefed animals 116, 478, and 0 DEGs were identified at FR2, FR8, and FR20 timepoints versus baseline respectively (Supplementary File 2). Observed gene expression changes in fasted animals was far more extensive as 1442, 1460, and 1215 DEGs were identified at FR2, FR8 and FR20 timepoints versus baseline respectively (Supplementary File 3). Table 1 summarizes the DEGs for all comparisons (FDR<0.05).

**Table 1:** Number of differentially expressed genes between CPF and FS animals and between timepoints within groups.

Comparison	Differentially Expressed Genes
CPFvFS, B	68
CPFvFS, FR2vB	13
CPFvFS, FR8vB	29
CPFvFS, FR20vB	30
FR2vB, CPF	116
FR8vB, CPF	478
FR20vB, CPF	0
FR2vB, FS	1442
FR8vB, FS	1460
FR20vB	1215

Abbreviations: CPF (Carbohydrate Prefed), FS (Fasted), B (Baseline), FR2, FR8 and FR20 (2, 8 and 20 hours after full resuscitation).

### Functional Analysis – Fed State Expression

**Figure 2:**
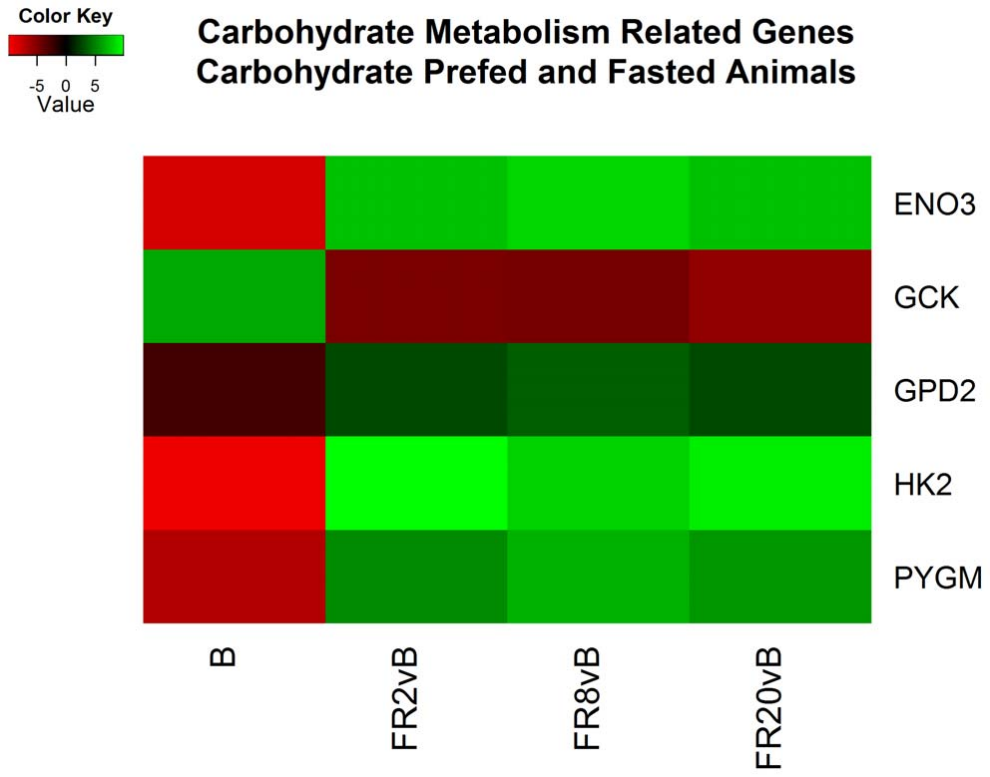
Heatmap of log2 fold changes of genes associated with carbohydrate metabolism between carbohydrate prefed (CPF) and fasted (FS) animals. Rows are differentially expressed genes (DEGs) following RNA sequencing. Columns denote the respective timepoints Baseline (B), 2 hours full resuscitation change from Baseline (FR2vB), 8 hours full resuscitation change from Baseline (FR8vB), and 20 hours full resuscitation change from Baseline (FR20vB). Green denotes higher concentration or larger changes in mRNA concentration in CPF whereas red denotes the opposite.

**Figure 3:**
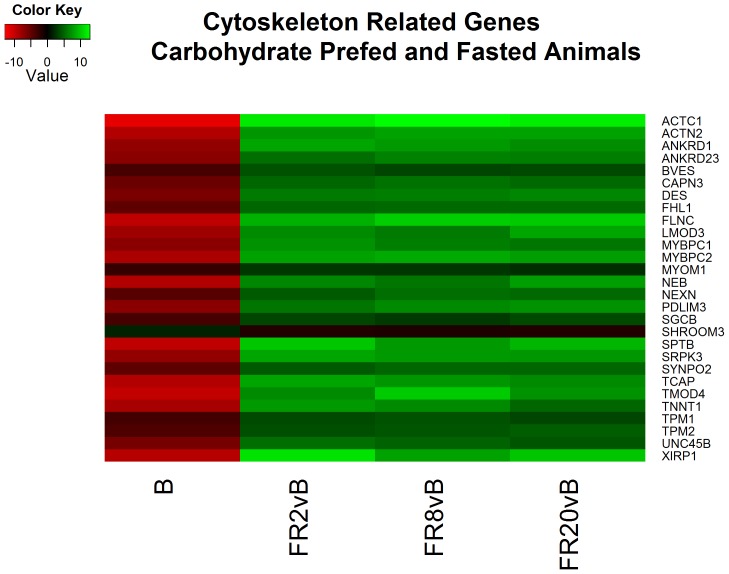
Heatmap of log2 fold changes of genes associated with cytoskeleton processes between carbohydrate prefed (CPF) and fasted (FS) animals. Rows are differentially expressed genes (DEGs) following RNA sequencing. Columns denote the respective timepoints Baseline (B), 2 hours full resuscitation change from Baseline (FR2vB), 8 hours full resuscitation change from Baseline (FR8vB), and 20 hours full resuscitation change from Baseline (FR20vB). Green denotes higher concentration or larger changes in mRNA concentration in CPF whereas red denotes the opposite.

Analysis of the differentially expressed transcripts using the Database for Annotation, Visualization and Integrated Discovery (DAVID) [19] and accompanying literature searches provided an overall picture of the overrepresented functions between the two fed states at various time points (Supplementary File 4). As expected, comparisons at baseline provide evidence of upregulation of transcripts important for carbohydrate metabolism in CPF animals (Figure 2). Curiously, the majority of the differences were related to contractile proteins associated with the cytoskeleton. In contrast to previous cell-based studies our in vivo investigation reports higher actin mRNA in fasted animals at baseline [22]–[24]. This trend reverses during FR2, FR8 and FR20 where cytoskeletal proteins in CPF exceed FS animals (Figure 3).

### Functional Analysis – Responses to Shock and Resuscitation within each Fed State

#### Carbohydrate prefed - FR2 v. baseline

Analysis of each timepoint relative to baseline within CPF animals revealed several responses that are characteristic of hemorrhagic shock and resuscitation. Analysis of FR2vB identified functional responses including increases in processes associated with cell adhesions, hormone responses and cell membrane processes (Supplementary File 5). Exploration of the genes within these categories revealed activation of gene expression related to evidence of wound healing, and apoptosis.

#### Carbohydrate prefed - FR8 v. baseline

**Figure 4:**
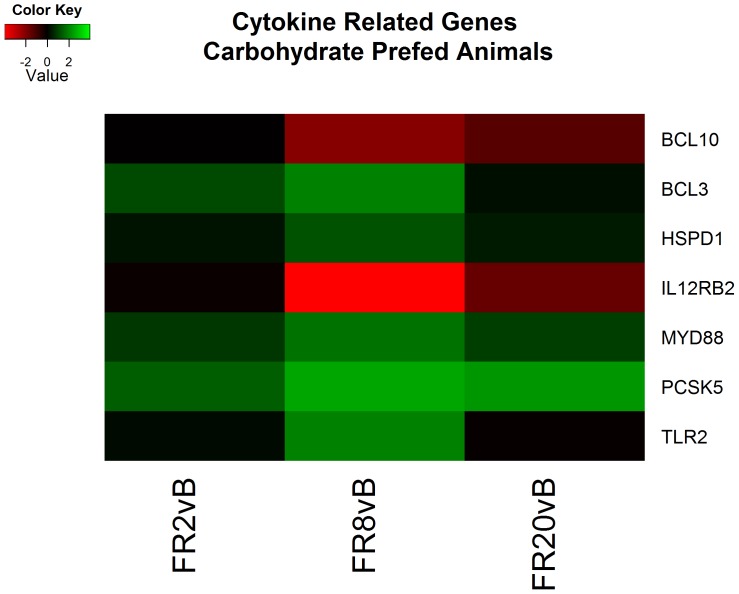
Heatmap of log2 fold changes of genes associated with cytokine related genes in carbohydrate prefed (CPF) animals at each resuscitation timepoint (2, 8, and 20 hours) relative to Baseline (B). Rows are differentially expressed genes (DEGs) following RNA sequencing. Columns denote the respective timepoints Baseline (B), 2 hours full resuscitation change from Baseline (FR2vB), 8 hours full resuscitation change from Baseline (FR8vB), and 20 hours full resuscitation change from Baseline (FR20vB). Green denotes increased mRNA expression with respect to Baseline whereas red denotes the opposite.

**Figure 5:**
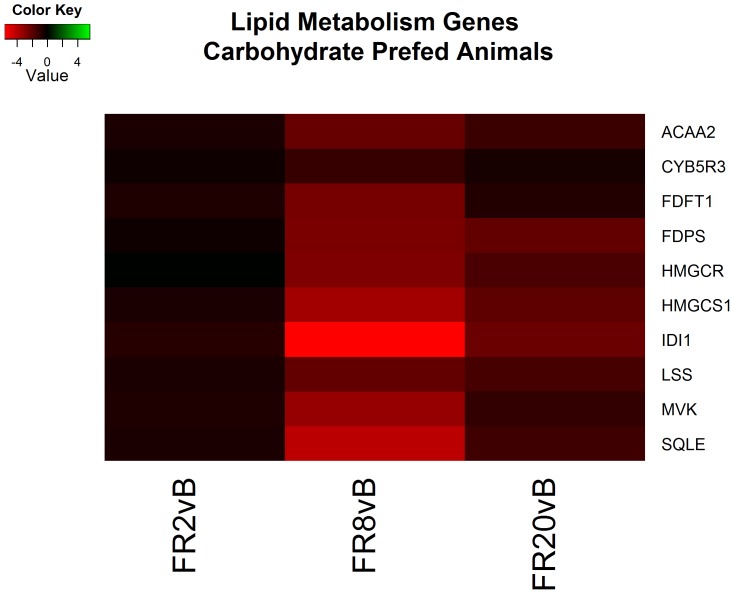
Heatmap of log2 fold changes of genes associated with lipid metabolism in carbohydrate prefed (CPF) animals at each resuscitation timepoint (2, 8, and 20 hours) relative to Baseline (B). Rows are differentially expressed genes (DEGs) following RNA sequencing. Columns denote the respective timepoints Baseline (B), 2 hours full resuscitation change from Baseline (FR2vB), 8 hours full resuscitation change from Baseline (FR8vB), and 20 hours full resuscitation change from Baseline (FR20vB). Green denotes increased mRNA expression with respect to Baseline whereas red denotes the opposite.

**Figure 6:**
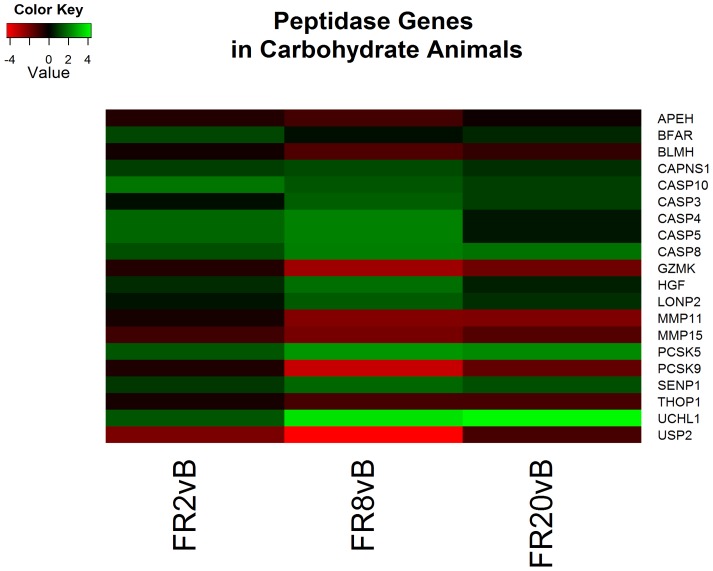
Heatmap of log2 fold changes of genes associated with peptidase activity in carbohydrate prefed (CPF) animals at each resuscitation timepoint (2, 8, and 20 hours) relative to Baseline (B). Rows are differentially expressed genes (DEGs) following RNA sequencing. Columns denote the respective timepoints Baseline (B), 2 hours full resuscitation change from Baseline (FR2vB), 8 hours full resuscitation change from Baseline (FR8vB), and 20 hours full resuscitation change from Baseline (FR20vB). Green denotes increased mRNA expression with respect to Baseline whereas red denotes the opposite.

Continued resuscitation in CPF animals continued to identify gene expression characteristic of the response to hemorrhagic shock and resuscitation. Functional clusters identified included altered cytokine production (Figure 4), lipid metabolism (Figure 5), oxidation-reduction processes, hormone responses, and peptidase activity (Figure 6) (Supplementary File 5). Genes associated with cytokine production included heat shock 60 kDa protein 1 (HSPD1) and myeloid differentiation primary response 88 (MyD88) which both induce pro-inflammatory cytokines [25]–[27]. Analysis of specific genes within categories suggested decreased cholesterol synthesis, decreased fatty acid beta-oxidation and apoptosis. All processes are typical following ischemia-reperfusion [28], [29]. No genes were found to be significant when comparing FR20 to baseline, possibly a consequence of the small sample size in the CPF group.

#### Fasted – FR2 v. baseline

Functional analysis of resuscitation in FS animals returned 37 clusters of similar processes to CPF animals including cell adhesions, wound healing, cytokine inflammation and apoptosis (Supplementary File 6). In contrast to CPF animals, cytoskeleton reorganization appears to be taking place wherein cytoskeletal genes are decreasing (Figure 3). This finding is concordant with previous reports of cytoskeleton destabilization following reperfusion [30].

#### Fasted – FR8 v. baseline

**Figure 7:**
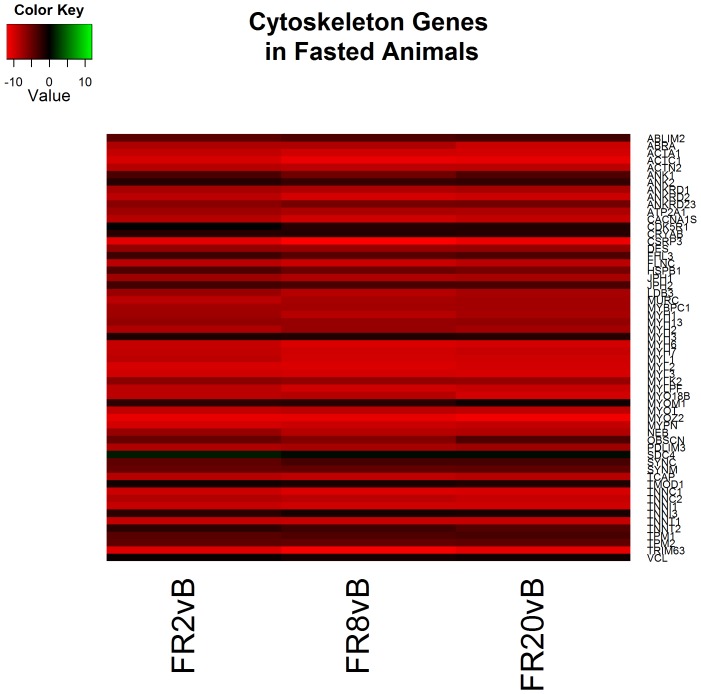
Heatmap of log2 fold changes of genes associated with cytoskeleton processes in fasted (FS) animals at each resuscitation timepoint (2, 8, and 20 hours) relative to Baseline (B). Rows are differentially expressed genes (DEGs) following RNA sequencing. Columns denote the respective timepoints Baseline (B), 2 hours full resuscitation change from Baseline (FR2vB), 8 hours full resuscitation change from Baseline (FR8vB), and 20 hours full resuscitation change from Baseline (FR20vB). Green denotes increased mRNA expression with respect to Baseline whereas red denotes the opposite.

**Figure 8:**
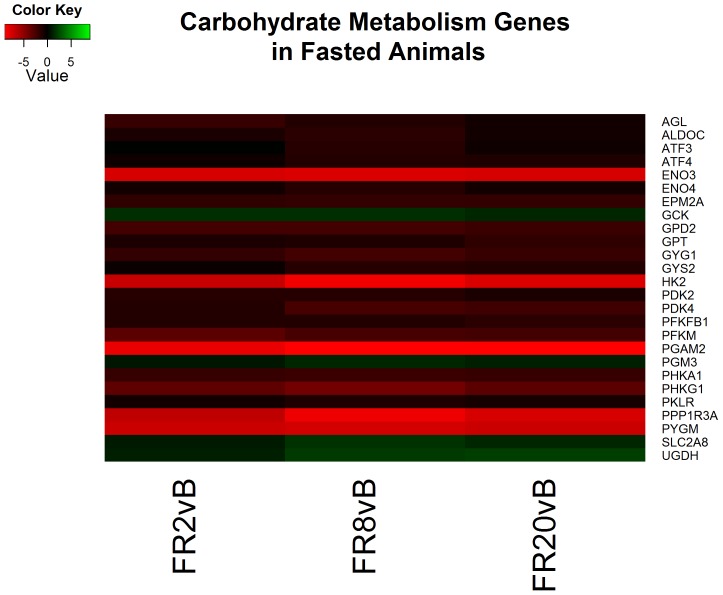
Heatmap of log2 fold changes of genes associated with carbohydrate metabolism in fasted (FS) animals at each resuscitation timepoint (2, 8, and 20 hours) relative to Baseline (B). Rows are differentially expressed genes (DEGs) following RNA sequencing. Columns denote the respective timepoints Baseline (B), 2 hours full resuscitation change from Baseline (FR2vB), 8 hours full resuscitation change from Baseline (FR8vB), and 20 hours full resuscitation change from Baseline (FR20vB). Green denotes increased mRNA expression with respect to Baseline whereas red denotes the opposite.

Following 8 hours of resuscitation FS, functional analysis identified 45 clusters that were very similar to CPF animals. The processes include cytokine inflammation, decreased cholesterol synthesis, and apoptosis (Supplementary File 6). In addition, cytoskeleton reorganization continues to take place as noted by decreasing cytoskeletal gene expression including actin and multiple elevated myosin heavy and light chains (MYH1, MYH2, MY3, MY6, MY7, MY13, MYL1, MYL2, MYL3) (Figure 7). In addition, glycolysis appears to be downregulated as several glycolytic genes decreased (HK2, PGAM2, ENO3, GPD2, PDK2, PFKM, and PKLR) (Figure 8).

#### Fasted – FR20 v. baseline

**Figure 9:**
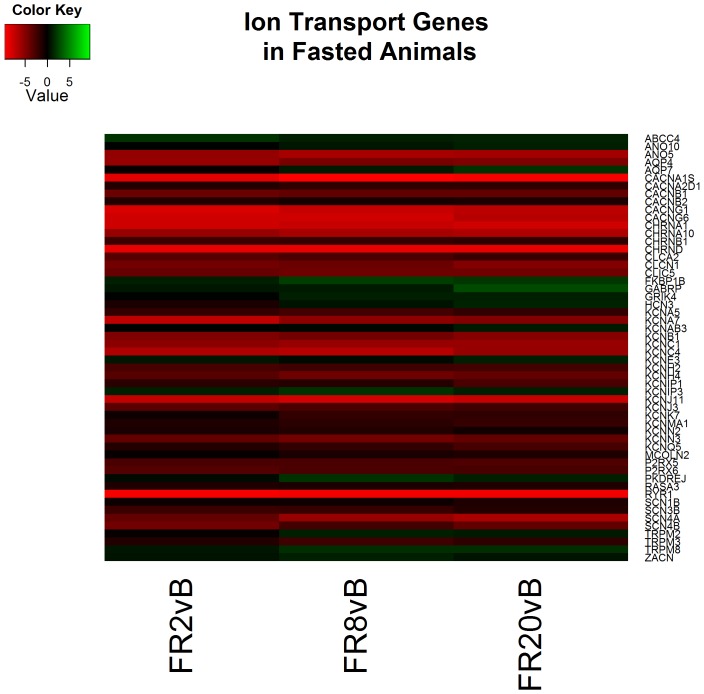
Heatmap of log2 fold changes of genes associated with ion transport in fasted (FS) animals at each resuscitation timepoint (2, 8, and 20 hours) relative to Baseline (B). Rows are differentially expressed genes (DEGs) following RNA sequencing. Columns denote the respective timepoints Baseline (B), 2 hours full resuscitation change from Baseline (FR2vB), 8 hours full resuscitation change from Baseline (FR8vB), and 20 hours full resuscitation change from Baseline (FR20vB). Green denotes increased mRNA expression with respect to Baseline whereas red denotes the opposite.

**Figure 10:**
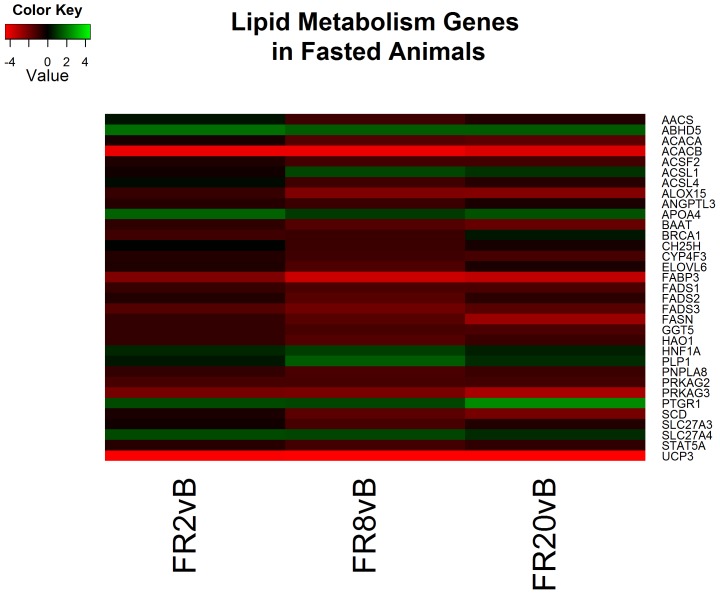
Heatmap of log2 fold changes of genes associated with carbohydrate metabolism in fasted (FS) animals at each resuscitation timepoint (2, 8, and 20 hours) relative to Baseline (B). Rows are differentially expressed genes (DEGs) following RNA sequencing. Columns denote the respective timepoints Baseline (B), 2 hours full resuscitation change from Baseline (FR2vB), 8 hours full resuscitation change from Baseline (FR8vB), and 20 hours full resuscitation change from Baseline (FR20vB). Green denotes increased mRNA expression with respect to Baseline whereas red denotes the opposite.

Lastly, of the 41 clusters identified by functional analysis following 20 hours of resuscitation primary processes continued to include cytoskeleton reorganization in addition to ion transport and glucose metabolism processes (Supplementary File 6). Genes associated with glycolytic and ion transport processes continue to both be decreased relative to baseline (Figures 8 & 9). Cholesterol and lipid oxidation are also decreased in overall expression (Figure 10).

## Discussion

We report that the primary differences between the liver transcriptomic response to hemorrhagic shock between fasted and carbohydrate prefed pigs during the first 24 hours in a polytrauma model of hemorrhagic shock and resuscitation consist of glucose metabolism and cytoskeletal remodeling. Consistent with known changes associated with ischemia and reperfusion, we also found evidence of cytokine activation, apoptosis, and lipid metabolism ontological categories. Additionally, genes associated with increased pro-inflammatory cytokines were elevated in CPF animals relative to FS animals. Our results suggest the anti-inflammatory state of the FS animals may be a result of the fasting metabolic state.

### Gene Ontology Profiles

We present the first, to our knowledge, comprehensive exploration of the pig liver transcriptome following a polytrauma model of hemorrhagic shock and resuscitation via RNA-seq. As expected following a carbohydrate prefed genes associated with carbohydrate metabolism were upregulated as indicated by elevated Glucokinase (GCK) and glycogen phosphorylase (PYGM) in CPF and FS animals respectively reflecting the alternate metabolic state wherein fasted animals are releasing glucose and CPF animals are breaking down available glucose.

The difference in cytoskeleton related genes in FS animals compared to CPF and over time suggests a link between fed state and metabolic response to hemorrhagic shock and resuscitation. Prior *in vitro* investigations have reported that glucagon, which is elevated during fasting periods, causes cellular shrinkage as a result of altered ion fluxes [31], [32]. Conversely, elevated insulin results in cellular swelling. Additionally, glucagon stimulates the transcription of actin followed by assembly of actin filaments [22]–[24]. Contrary to these *in vitro* studies, however, our *in vivo* investigation reports increased actin transcription in fasted animals compared to CPF animals. This cellular shrinkage has been previously described as triggering the ‘catabolic state’ of a cell resulting in a multitude of altered processes [33]. The presence of this cytoskeleton remodeling is further supported in our study by upregulation of contractile proteins including multiple myosins for intracellular transport. In addition, cellular swelling stabilizes actin filaments [34] which helps explain why cytoskeleton mRNA expression is higher in CPF than FS during resuscitation which usually results in actin depolymerization [35].

Previous studies elucidating the impact of cell swelling during ischemia and reperfusion report that cells naturally become hyperosmotic during ischemia and consequently take up additional fluid [36], [37]. This increased swelling and resultant cellular edema may compress capillaries thereby reducing or preventing reperfusion of capillary beds [38]–[40]. This process results in zones that prevent reperfusion resulting in the ‘no-reflow’ phenomenon [41]. It is possible that the fasting state displays a shrunken catabolic state that may provide an initial condition to partially mitigate the subsequent swelling that is an unavoidable consequence of ischemia and reperfusion. The benefit of induced cellular shrinkage on hepatocellular injury has been shown previously whereby hypertonic preconditioning (i.e. induced cellular shrinkage) reduces hepatocellular damage following ischemia reperfusion [42]. Furthermore, the use of hypertonic saline for reperfusion has been shown to decrease susceptibility to sepsis, mitigate inflammation, and reduce apoptosis after hemorrhagic shock [43]–[45] in addition to being attractive for small-volume resuscitation development [4].

**Figure 11:**
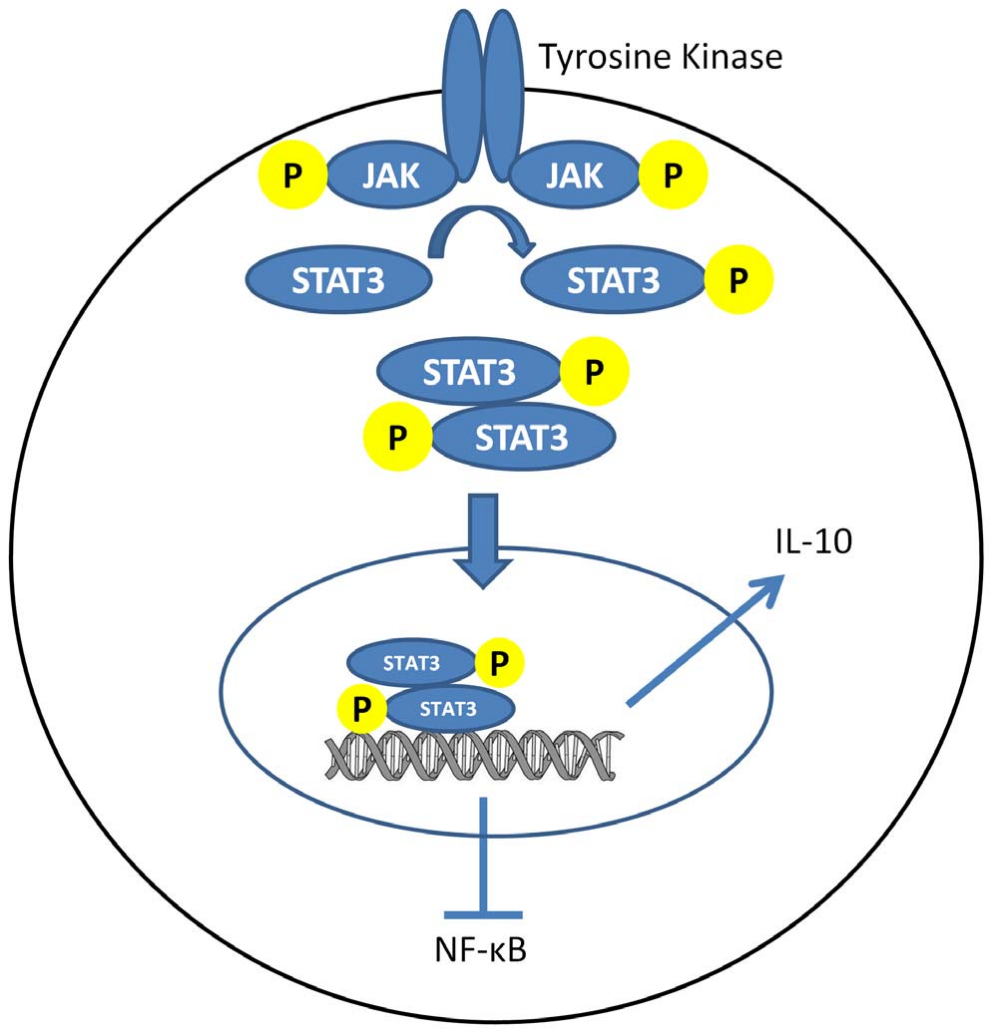
Diagram of simplified Interleukin-10 (IL-10) pathway. A hyperosmotic condition induces cellular shrinkage and activates tyrosine phosphorylation of Janus kinases (JAK) to subsequently phosphorylate STAT3 and IL-10 production.

The full molecular mechanisms behind the proposed state are not fully elucidated however several components are understood. One direct result of cellular shrinkage is vasodilation potentially mediating the no-reflow condition. Furthermore, cellular shrinkage is a consequence of hypertonic treatments which have been shown mitigate inflammation following shock [46], [47]. The hyperosmotic environment causes cellular shrinkage which activates the tyrosine phosphorylation of Janus kinases. These kinases activate the transcription factor STAT3 [48] which ultimately stimulates Interleukin 10 (IL-10) production while simultaneously inhibiting pro-inflammatory cytokines (e.g. IL-1β, TNF-α, and intercellular adhesion molecules) by inhibiting the transcription factor NF-κB [49], [50] (Figure 11). Although IL-10 was not individually identified, the anti-inflammatory state of the FS is supported as several pro-inflammatory genes are elevated in CPF animals (Figure 8). Further research on the anti-inflammatory properties of cellular shrinkage is required.

Although this study utilized a larger sample size, our groups were unbalanced (18 versus 5). This difference in group size may have partially biased estimates. However, given the quality of the RNA-seq, consistency across timepoints, and agreement with supporting literature supports our results. Lastly, it is important to recall that the liver is only one component of the overall systemic response to hemorrhagic shock and resuscitation. Analysis of other organs and tissues would be should be pursued.

## Conclusion

Analysis of the liver transcriptome between carbohydrate prefed and fasted pigs following hemorrhagic shock and resuscitation reveals that the first 24 hours results in different changes to the cytoskeleton structure. Our results suggest this both a metabolic response to decreased carbohydrate substrates and structural modifications in response to cellular shrinkage. Further evidence suggests that this shrunken state provides an anti-inflammatory condition that may decrease hepatocellar damage. More work is required to investigate the potential benefits of alternative diets such as the anti-inflammatory effects of increased intake of omega-3 fatty acids.

## Supporting Information

File S1 List of genes differentially expressed between Carbohydrate Prefed and Fasted animals at Baseline and FR2, FR8 and FR20 vs. Baseline.
(XLSX)

File S2 List of genes differentially expressed over time within Carbohydrate Prefed animals.
(XLSX)

File S3 List of genes differentially expressed over time within Fasted animals.
(XLSX)

File S4 List of gene ontology profiles comparing Carbohydrate Prefed and Fasted animals at Baseline and FR2, FR8 and FR20 vs. Baseline.
(XLSX)

File S5 List of gene ontology profiles over time within Carbohydrate Prefed animals at Baseline and FR2, FR8 and FR20 vs. Baseline.
(XLSX)

File S6 List of gene ontology profiles over time within Fasted animals at Baseline and FR2, FR8 and FR20 vs. Baseline.
(XLSX)

File S7 R code for differential expression analysis.
(R) Click here for additional data file.
